# Squamosamide Derivative FLZ Protects Retinal Pigment Epithelium Cells from Oxidative Stress through Activation of Epidermal Growth Factor Receptor (EGFR)-AKT Signaling

**DOI:** 10.3390/ijms151018762

**Published:** 2014-10-17

**Authors:** Li-Bo Cheng, Chun-Ming Chen, Hong Zhong, Li-Juan Zhu

**Affiliations:** Eye Department, Li-yang City Hospital of Traditional Chinese Medicine, Li-Yang City 213300, China; E-Mails: chunmingchenapp@163.com (C.-M.C.); hongapple126@126.com (H.Z.)

**Keywords:** age-related macular degeneration (AMD), retinal pigment epithelium (RPE), squamosamide derivative FLZ, apoptosis, AKT signaling and EGFR (epidermal growth factor receptor) trans-activation

## Abstract

Reactive oxygen species (ROS)-mediated retinal pigment epithelium (RPE) cell apoptosis is attributed to age-related macular degeneration (AMD) pathogenesis. FLZ, a novel synthetic squamosamide derivative from a Chinese herb, *Annona glabra*, has displayed significant cyto-protective activity. In the current study, we explored the pro-survival effect of FLZ in oxidative stressed-RPE cells and studied the underlying signaling mechanisms. Our results showed that FLZ attenuated hydrogen peroxide (H_2_O_2_)-induced viability decrease and apoptosis in the RPE cell line (ARPE-19 cells) and in primary mouse RPE cells. Western blotting results showed that FLZ activated AKT signaling in RPE cells. The AKT-specific inhibitor, MK-2206, the phosphoinositide 3-kinase (PI3K)/AKT pan inhibitor, wortmannin, and AKT1-shRNA (short hairpin RNA) depletion almost abolished FLZ-mediated pro-survival/anti-apoptosis activity. We discovered that epidermal growth factor receptor (EGFR) trans-activation mediated FLZ-induced AKT activation and the pro-survival effect in RPE cells, and the anti-apoptosis effect of FLZ against H_2_O_2_ was inhibited by the EGFR inhibitor, PD153035, or by EGFR shRNA-knockdown. In conclusion, FLZ protects RPE cells from oxidative stress through activation of EGFR-AKT signaling, and our results suggest that FLZ might have therapeutic values for AMD.

## 1. Introduction

Age-related macular degeneration (AMD) is a progressive retinal degeneration disease, which causes blindness among elderly people [1]. Ultraviolet (UV) exposure and reactive oxygen species (ROS) damage are the main pathological causes of AMD [2,3]. Under oxidative stress, reactive free radicals, including superoxide, hydroxyl radical, singlet oxygen and hydrogen peroxide (H_2_O_2_), induce damage to retinal pigment epithelium (RPE) cells by excessively oxidizing key cellular components [2,3]. Anti-oxidants or zinc-containing supplements could reduce AMD progression in human [4,5]. Thus, oxidative stress prevention is an effective strategy to slow down or even reverse AMD progression. Groups including ours have been adding H_2_O_2_ to cultured RPE cells to create a cellular model of AMD and to explore the potential interfering strategies [6,7,8,9,10].

FLZ (chemical name *N*-[2-(4-hydroxy-phenyl)-ethyl]-2-(2,5-dimethoxy-phenyl)-3-(3-methoxy-4-hydroxy-phenyl)-acrylamide) is a synthetic novel derivative of squamosamide found in the Chinese herb, *Annona glabra* [11,12,13,14,15,16]. Studies have shown that FLZ displayed a significant neuroprotective effect both *in vivo* and *in vitro* [11,12,13,14,15,16]. Further, FLZ exerted dramatic myocardial protection activity [14,17]. The underling mechanism of the FLZ-induced cytoprotective effect has not yet been fully understood, although AKT activation has been proposed [11,13]. AKT plays a vital role in cell survival [18]. AKT regulates multiple downstream targets to prevent cell apoptosis [18]. Activated AKT suppresses apoptosis by phosphorylating and inactivating the pro-apoptotic proteins (*i.e.*, Bad and caspase 9). Further, AKT activates nuclear factor-kappa B (NF-κB) to inhibit cell apoptosis [19,20]. Our previous study demonstrated that nerve growth factor (NGF) and α-melanocyte stimulating hormone (α-MSH) rescued oxidative stressed-RPE cells by activating AKT signaling [6].

In light of this information, we proposed that FLZ might exert a protective effect against oxidative stress in RPE cells. We thus explored the potential role of FLZ on H_2_O_2_-treated RPE cells. We identified a new FLZ-mediated pro-survival pathway that attenuated H_2_O_2_-induced RPE cell damage and that may minimize the risk of developing AMD.

## 2. Results and Discussion

### 2.1. FLZ Protects Retinal Pigment Epithelium (RPE) Cells from Hydrogen Peroxide (H_2_O_2_)

**Figure 1:**
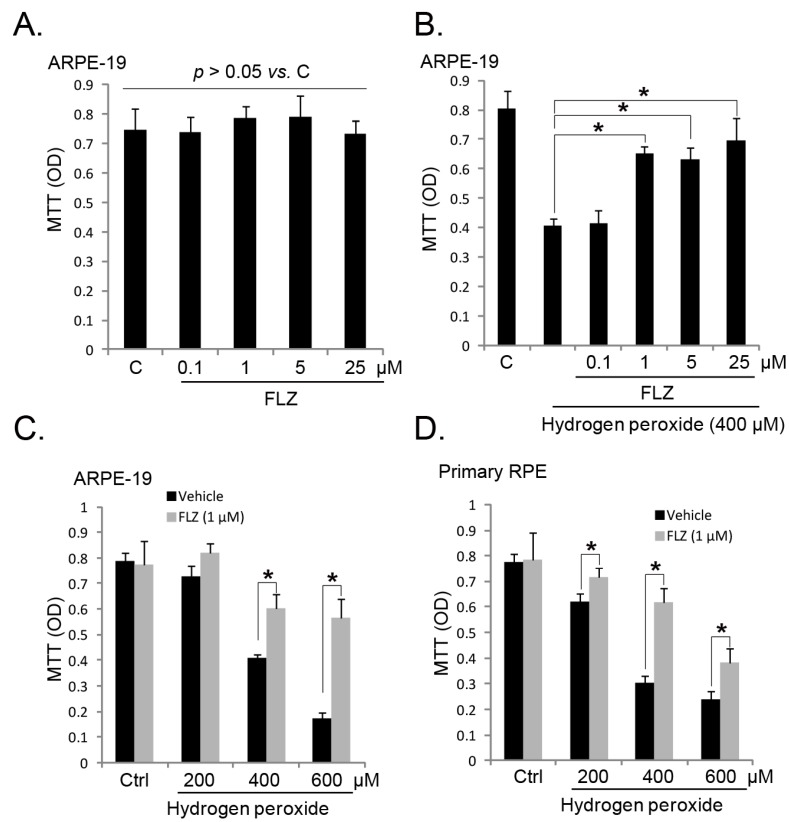
FLZ protects RPE (retinal pigment epithelium) cells from hydrogen peroxide (H_2_O_2_). The cell viability of APRE-19 cells with the indicated H_2_O_2_ or plus FLZ treatment for 24 h was tested by the MTT (3-(4,5-dimethyl-2-thiazolyl)-2,5-diphenyl-2-H-tetrazolium bromide) assay (**A**–**C**); Primary cultured mouse RPE cells were treated with H_2_O_2_ (200–600 μM) with or without FLZ (1 μM) for 24 h; cell viability was tested by the MTT assay (**D**). Experiments were repeated three times to ensure the consistency of the results. “C” stands for the untreated control group. “Ctrl” stands for no H_2_O_2_. Vehicle stands for 0.1% dimethyl sulfoxide (DMSO). * *p <* 0.05 (Analysis of Variance, ANOVA).

In the current study, we aimed to understand the potential role of FLZ against oxidative stress in RPE cells. MTT (3-(4,5-dimethyl-2-thiazolyl)-2,5-diphenyl-2-H-tetrazolium bromide) cell viability results in Figure 1A demonstrated that FLZ (0.1–25 μM) alone had no detectable effect on APRE-19 (a human RPE cell line) survival (*p* > 0.05 *vs.* untreated control group). Significantly, FLZ at doses of 1–25 μM attenuated H_2_O_2_-induced APRE-19 cell viability decrease (Figure 1B,C). Further, in primary cultured RPE cells, FLZ (1 μM) also inhibited H_2_O_2_-induced cell viability reduction (Figure 1D). FLZ showed the most significant cyto-protective activity when H_2_O_2_ was at a concentration of 400 μM, and this concentration was chosen for further experiments. Thus, these results show that FLZ protects RPE cells against H_2_O_2_.

### 2.2. FLZ Attenuates H_2_O_2_-Induced RPE Cell Apoptosis

**Figure 2:**
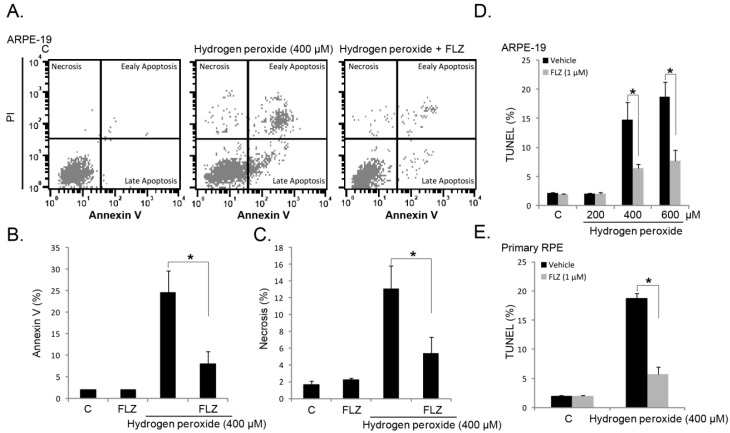
FLZ attenuates H_2_O_2_-induced RPE cell apoptosis. Annexin-V/PI FACS images of APRE-19 cells treated with H_2_O_2_ (400 μM) or plus FLZ (1 μM) for 24 h (**A**); results were quantified in (**B**,**C**); APRE-19 cells (**D**) and primary mouse RPE cells (**E**) were treated with the indicated concentration of H_2_O_2_ or plus FLZ (1 μM) for 24 h, and cell apoptosis was analyzed by the terminal deoxynucleotidyl transferase dUTP nick end labeling (TUNEL) staining assay. Experiments were repeated three times to ensure the consistency of the results. “C” stands for the untreated control group. Vehicle stands for 0.1% DMSO. * *p <* 0.05 (ANOVA).

Next, we tested the role of FLZ on RPE cell apoptosis induced by H_2_O_2_. RPE cell apoptosis was tested by the Annexin-V FACS assay and the terminal deoxynucleotidyl transferase dUTP nick end labeling (TUNEL) staining assay [8,21]. In APRE-19 cells, apoptosis was induced by H_2_O_2_ (Figure 2A,B,D). Co-treatment with FLZ (1 μM) significantly inhibited H_2_O_2_-induced APRE-19 cell apoptosis (Figure 2A,B,D). Interestingly, we noticed a proportion of necrotic APRE-19 cells (*Annexin-V*^−/−^ and *PI*^+/+^ cells) after H_2_O_2_ stimulation, which was also inhibited by FLZ co-treatment (Figure 2A,C). In primary RPE cells, H_2_O_2_-induced apoptosis was again alleviated by FLZ (Figure 2E). Together, these results demonstrate that FLZ attenuates H_2_O_2_-induced RPE cell apoptosis.

### 2.3. FLZ Activates AKT in RPE Cells

**Figure 3:**
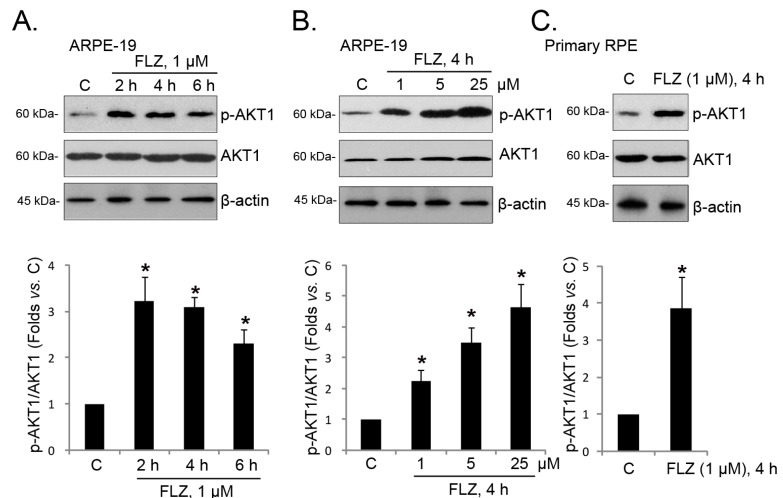
FLZ activates AKT in RPE cells. APRE-19 cells (**A**,**B**) and primary mouse RPE cells (**C**) were treated with FLZ for the indicated time, and phospho-(p-) AKT1 (Ser 473), regular AKT1 and β-actin were tested by western blotting. AKT1 phosphorylation was quantified. Experiments were repeated three times to insure consistency of results. “C” stands for the untreated control group. * *p <* 0.05 *vs.* Group “C” (ANOVA).

Next, we explored the mechanisms underlying the pro-survival effect of FLZ by focusing on AKT signaling. FLZ is shown to activate AKT in other cells [11], and AKT signaling is essential for the survival of RPE cells [18]. Western blotting results in Figure 3A,B demonstrated that FLZ induced AKT activation in both time- and dose-dependent manners. AKT activation was seen as early as two hours after FLZ treatment, and it lasted as least for six hours (Figure 3A). Further, as shown in Figure 3C, AKT was also activated by FLZ in primary RPE cells. Together, these results confirm AKT activation by FLZ in RPE cells.

### 2.4. AKT Activation Mediates the FLZ-Induced Pro-Survival Effect against H_2_O_2_

**Figure 4:**
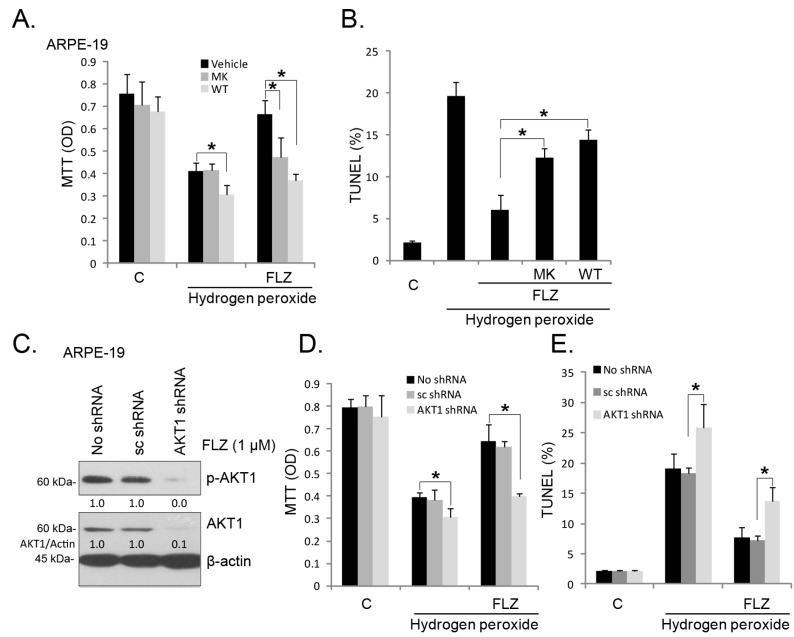
AKT activation mediates the FLZ-induced pro-survival effect against H_2_O_2_. APRE-19 cells were treated with H_2_O_2_ (400 μM) or plus FLZ (1 μM), in the presence of or absence of MK-2206 (MK, 5 μM) and wortmannin (WT, 5 μM) for 24 h; cell viability was tested by the MTT assay (**A**); and cell apoptosis was tested by the TUNEL staining assay (**B**). The control APRE-19 cells (no shRNA infection) or stable APRE-19 cells infected with scramble-shRNA (sc shRNA) or AKT1-shRNA were treated with H_2_O_2_ (400 μM) or plus FLZ (1 μM) for 24 h, cell viability was tested by the MTT assay (**D**), and apoptosis was tested by the TUNEL staining assay (**E**); FLZ (1 μM)-induced AKT1 phosphorylation was tested (4 h) and AKT1 phosphorylation and expression (*vs.* β-actin) were quantified (**C**). Experiments were repeated three times to ensure the consistency of the results. “C” stands for the untreated control group. Vehicle stands for 0.1% DMSO. * *p <* 0.05 (ANOVA).

Our group has shown that a number of agents, including NGF [6], ginsenoside Rg-1 [21], salvianolic acid A [8] and α-MSH [9], induce the pro-survival effect in RPE cells, which is mediated, at least in part, by AKT activation. The results above have shown that FLZ activated AKT in RPE cells. Next, we explored the role of AKT activation in the pro-survival effect. As shown in Figure 4A,B, in APRE-19 cells, the AKT-specific inhibitor, MK-2206 (MK) [22,23], and the phosphoinositide 3-kinase (PI3K)/AKT pan inhibitor, wortmannin (WT) [24], largely inhibited the pro-survival and anti-apoptosis activities of FLZ against H_2_O_2_, indicating that AKT activation is required for FLZ’s effect. To further support this hypothesis, we utilized targeted shRNA to selectively knockdown AKT1 in ARPE-19 cells (Figure 4C), and the results showed that FLZ-induced pro-survival and anti-apoptosis activities against H_2_O_2_ were diminished when AKT was depleted by shRNA (Figure 4D,E). Thus, AKT activation is important for the FLZ-mediated survival effect in RPE cells.

### 2.5. EGFR (Epidermal Growth Factor Receptor) Trans-Activation Mediates FLZ-Induced AKT Activation and Pro-Survival Effect in RPE Cells

**Figure 5:**
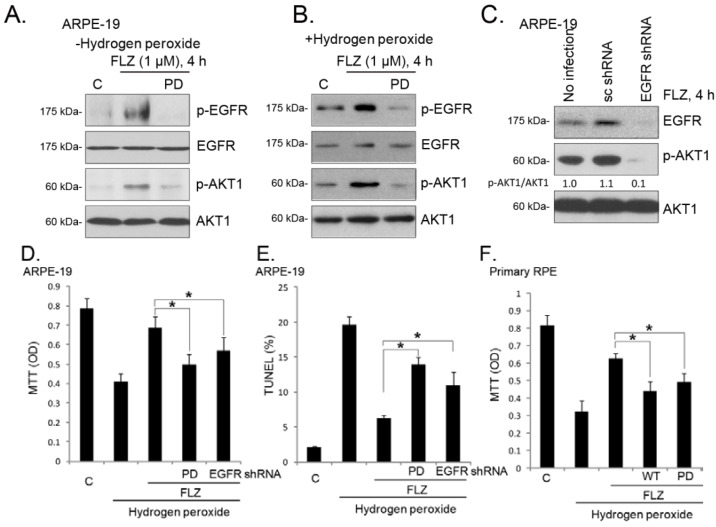
EGFR (epidermal growth factor receptor) trans-activation mediates FLZ-induced AKT activation and the pro-survival effect in RPE cells. APRE-19 cells were pre-treated with EGFR inhibitor PD153035 (PD, 1 μM), followed by FLZ (1 μM) stimulation in the presence or absence of H_2_O_2_ (400 μM); cells were further cultured for four hours, and EGFR, p-EGFR (Tyr 1068), AKT1 and p-AKT1 (Ser 473) were tested by western blotting (**A**,**B**); The control APRE-19 cells (no infection) or APRE-19 cells infected with scramble-shRNA or EGFR-shRNA lentiviral particles (20 μL/mL, 24 h infection) were treated with FLZ (1 μM) for 4 h, and EGFR, AKT1 and p-AKT1 (Ser 473) were tested by western blotting (**C**); p-AKT1 was quantified. APRE-19 cells were treated with H_2_O_2_ (400 μM) or plus FLZ (1 μM), in the presence or absence of PD153035 (PD, 1 μM) or EGFR-shRNA lentiviral particles (20 μL/mL, 24 h infection), and cells were further cultured for 24 h. The MTT assay (**D**) and the TUNEL staining assay (**E**) were performed to test cell viability and apoptosis, respectively. Primary mouse RPE cells were treated with H_2_O_2_ (400 μM) or plus FLZ (1 μM), in the presence or absence of PD153035 (PD, 1 μM) or wortmannin (WT, 5 μM); cell viability was tested by the MTT assay after 24 h (**F**). Experiments were repeated three times to ensure the consistency of the results. “C” stands for the untreated control group. * *p <* 0.05 (ANOVA).

**Figure 6:**
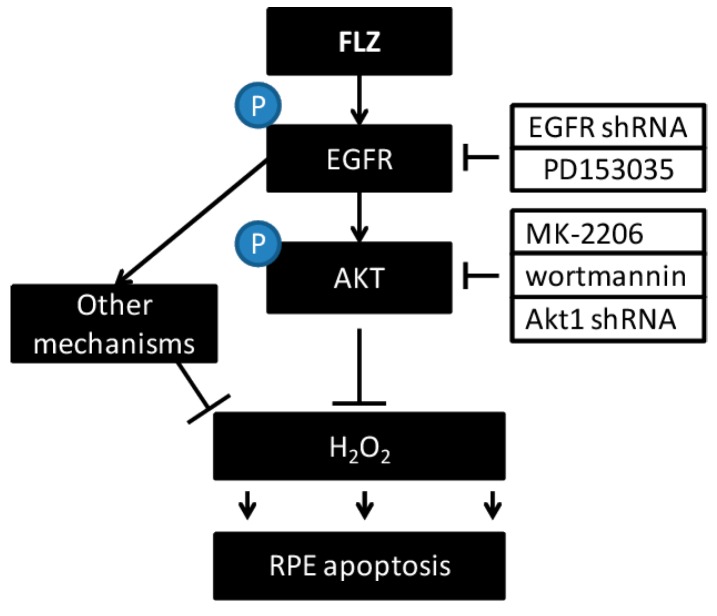
The proposed signaling pathway of this study.

Next, we studied the potential upstream signaling for AKT activation by FLZ in RPE cells. The EGF-EGFR signaling network is among one of the best-characterized signaling systems [25]. Besides being activated by its ligands, EGFR could also be activated indirectly by a number of agents, a process termed EGFR “trans-activation” [26,27,28,29,30]. Western blotting results in Figure 5A demonstrated that FLZ induced EGFR Tyr 1068 phosphorylation, the EGFR activation indicator, in cultured ARPE-19 cells, which was blocked by the EGFR inhibitor, PD153035 (PD) [31,32]. Meanwhile, PD153035 prevented FLZ-induced AKT activation (Figure 5A), indicating that EGFR trans-activation is required for FLZ-induced AKT activation. This is further supported by the fact that EGFR depletion by shRNA dramatically inhibited AKT activation by FLZ in APRE-19 cells (Figure 5C). H_2_O_2_ by itself could also slightly induce phosphorylation of EGFR and AKT in ARPE-19 cells, which was further increased by co-administration of FLZ (Figure 5B). Significantly, the EGFR inhibitor, PD153035, blocked EGFR-AKT phosphorylation by FLZ plus H_2_O_2_ (Figure 5A,B). These results suggest that trans-activation of EGFR and activation of its downstream signaling, AKT, are early events in H_2_O_2_-treated RPE cells (see the related reports [33,34,35]). FLZ increases AKT phosphorylation in H_2_O_2_-treated APRE-19 cells through enhancing EGFR activation. In APRE-19 cells, the pro-survival and anti-apoptosis activities of FLZ against H_2_O_2_ were alleviated by PD153035 or EGFR shRNA knockdown (Figure 5D,E). As shown in Figure 5F, in primary RPE cells, FLZ-induced pro-survival activity was similarly inhibited by PD153035 and wortmannin (WT). Together, we concluded that EGFR mediated FLZ-induced AKT activation and pro-survival activity in RPE cells (Figure 6).

### 2.6. Discussion

The vision loss among elderly AMD patients starts from abnormalities in the RPE, along with photoreceptor damage, Bruch’s membrane thickening and choriocapillary hypo-perfusion [36]. These are considered as the main characteristics of AMD [37]. UV radiation and subsequent oxidative stress damage are known as the main contributors to AMD [38,39,40,41,42,43]. In the current study, we showed that FLZ activated EGFR-AKT signaling and attenuated H_2_O_2_-induced RPE cell death and apoptosis. These effects by FLZ were inhibited by AKT/EGFR inhibition or depletion. Thus, we suggest that FLZ prevents H_2_O_2_-induced RPE cell damage through activating EGFR-AKT signaling (Figure 6).

EGFR is functionally expressed in RPE cells [44,45,46]. EGFR and their ligands modulate cellular functions in a variety of ways, including proliferation, survival, adhesion, migration and apoptosis resistance [47]. The EGFR ligands, including EGF, amphiregulin and transforming growth factor alpha (TGFα), as well as and heparin binding EGF (HB-EGF) and epiregulin directly bind to and activate EGFR [47]. Activated EGFR recruits adaptor proteins to activate downstream signaling molecules (*i.e.*, AKT signaling) [48] and to promote cell survival [47]. EGFR could also be trans-activated indirectly by various stimuli [26,27,28,29,30]. In the current study, we found that FLZ-induced AKT activation was also mediated through EGFR trans-activation. The conclusion was supported by the fact that EGFR inhibitor or silencing inhibited AKT activation by FLZ. Meanwhile, the pro-survival activity of FLZ against H_2_O_2_ was also attenuated by EGFR inhibitor or depletion. Thus, EGFR trans-activation is the upstream signal for FLZ-induced AKT activation, which promotes RPE cell survival (Figure 6).

## 3. Experimental Section

### 3.1. Chemical and Reagents

FLZ is a white powder with 99% purity and is insoluble in water [16]. FLZ was synthesized by Wuxi Ya-tai Bio Company (Wuxi, China) based on the structure described in [16]. FLZ was dissolved in dimethyl sulfoxide (DMSO) and added to the cell culture medium with a maximum DMSO concentration of 0.1%. PD153035, MK-2206 and wortmannin were purchased from Calbiochem (Darmstadt, Germany). The antibody against β-actin was purchased from Sigma (St. Louis, MO, USA). All other antibodies used in this study were obtained from Cell Signaling Tech (Danvers, MA, USA).

### 3.2. APRE-19 Cell Culture

As reported [7], the human retinal pigment epithelial cell line (ARPE-19) was routinely maintained in Dulbecco’s modified Eagle’s medium (DMEM)/Nutrient Mixture F-12 (DMEM/F12, Gibco, Carlsbad, CA, USA) containing 10% fetal bovine serum (FBS) (Hyclone, Shanghai, China), penicillin/streptomycin (1:100, Sigma, St. Louis, MO, USA) and 4 mM l-glutamine and 0.19% HEPES (hydroxyethyl piperazineethanesulfonic acid, Sigma), in a humidified incubator at 37 °C and 5% CO_2_.

### 3.3. Primary Mouse RPE Cell Isolation and Culture

As reported [7,9], C57/B6 mice at the age of 3–5 days were given anesthesia by 75% alcohol, and the eyeballs in asepsis were taken out and diluted several times with D-hank’s fluid. After soaking in the DMEM/F-12 for 6 h, the eyeballs were taken out, and the retinas were striped carefully. Parenzyme (0.125%) was added to digest for 20 min at 37 °C before adding culture medium containing blood serum to terminate digestion. Then, the supernatant was centrifuged twice at 1000 r/min in the culture medium (80% DMEM/F-12, 20% FBS) to produce a cell suspension after inoculation into the 75-cm^2^ culture flask. Cells were divided and were used for the designed experiments. For all experiments, RPE cells (primary and ARPE-19 cells) were serum-starved overnight using serum-free DMEM medium, and the next day, FLZ and inhibitors were added to the cells.

### 3.4. Cell Viability Assay

Cell viability was assessed by the 3-[4,5-dimethylthylthiazol-2-yl]-2,5 diphenyltetrazolium bromide (MTT) (Sigma, Shanghai, China) assay. In brief, RPE cells were collected and seeded in 96-well plates at a density of 1 × 10^5^ cells/well in 200 mL of culture medium. After treatment, 20 μL of MTT solution (5 mg/mL) were added to each well for 4 h at 37 °C, and cell viability was determined by measuring absorbance at 490 nm using a microplate spectrophotometer (Molecular Devices, Sunnyvale, CA, USA). The OD value was detected as an indicator of RPE cell viability.

### 3.5. Western Blotting

As reported [7,9], aliquots of 20 μg of proteins (lysed by 40 mM HEPES (pH 7.5), 120 mM NaCl, 1 mM EDTA (Ethylene Diamine Tetraacetic Acid), 10 mM pyrophosphate, 10 mM glycerophosphate, 50 mM NaF, 0.5 mM orthovanadate, EDTA-free protease inhibitors (Roche, Shanghai, China) and 1% Triton) were separated by 10% SDS (sodium dodecyl sulfate) polyacrylamide gel electrophoresis (SDS-PAGE) and transferred onto polyvinylidene difluoride (PVDF) membranes (Millipore, Bedford, MA, USA). After blocking with 10% non-fat dry milk for 1 h, membranes were incubated with the described antibodies overnight at 4 °C, followed by incubation with secondary antibodies for one hour at room temperature. The blots were visualized with enhanced chemiluminescence (ECL). Band intensities in the immunoblots were quantified by densitometry using ImageJ software (NIH, Bethesda, MD, USA). Phospho-kinases were always normalized to non-phospho-controls [7].

### 3.6. Annexin-V/PI FACS (Fluorescence-Activated Cell Sorting) Assay

RPE cell apoptosis was measured by Annexin-V fluorescence-activated cell sorting (FACS) according to the manufacturer’s protocol (Sigma). Briefly, after treatment, cells were washed twice with cold PBS (phosphate buffer solution) and incubated in 300 μL binding buffer containing 3 μL of Annexin-V-FITC (fluorescein isothiocyanate) and 3 μL of propidium iodine (PI) in the dark for 15 min at room temperature. The stained samples (containing 200,000 cell/sample) were then analyzed on a FACSCalibur flow cytometer within 1 h following the manufacturer’s protocol (Coulter, Hialeah, FL, USA). Annexin-V percentage was recorded as an indicator of apoptosis intensity; while *Annexin-V*^−/−^ and *PI*^+/+^ cells were labeled as necrotic cells. All experiments were performed in triplicate.

### 3.7. TUNEL (Terminal Deoxynucleotidyl Transferase dUTP Nick End Labeling) Staining

RPE cell apoptosis was detected by the TUNEL. *In Situ* Cell Death Detection Kit (Roche Molecular Biochemicals, Indianapolis, IN, USA), according to the manufacturer’s instructions. RPE cells were also stained with 4',6'-diamino-2-phenylin-dole (DAPI, blue fluorescence; Molecular Probes) to visualize the cell nuclei. The apoptosis rate was determined by TUNEL percentage, which was calculated by the number of TUNEL-positive cells divided by the number of DAPI-stained cells. At least 1000 total cells in 10 views from 10 repeat wells (1 × 100) of each condition were included for counting the TUNEL percentage.

### 3.8. Stable AKT1 Knockdown by Short Hairpin RNA (shRNA)

The lentiviral particles containing scramble shRNA or AKT1 shRNA were synthesized, verified and provided by Kaiji Biotech (Shanghai, China). The hairpin sequences used for AKT1 were as follows: forward, 5'-CCGGTGCTGCTTCCTCCTCAAGAATGTTCAAGAGACATTCTTGAGGAGGAAGTAGCTTTTTGGAAG-3'; reverse, 5'-AATTCTTCCAAAAAGCTACTTCCTCCTCAAGAATGTCTCTTGAACATTCTTGAGGAGGAAGCAGCA-3'. The lentiviral shRNA (20 μL/mL) was added to ARPE-19 cells for 24 h, and stable clones expressing shRNA were further selected by puromycin (1.0 μg/mL). Cell culture medium containing puromycin was renewed every 48 h, until resistant colonies could be identified (4–5 passages). The expression of AKT1 and the loading control (β-actin) in stable cells was tested.

### 3.9. Transit Knockdown of Epidermal Growth Factor Receptor (EGFR) by shRNA

The lentiviral particles containing scramble shRNA (sc-108080) or human EGFR shRNA (sc-29301-V) were purchased from Santa Cruz Biotech (Santa Cruz, CA, USA). Lentiviral shRNA particles (20 μL/mL) were added to the ARPE-19 cells for 24 h, and the expression level of EGFR and the loading control in infected cells were tested.

### 3.10. Statistical Analysis

All data were presented as the mean ± standard deviation (SD). Statistics were analyzed by one-way ANOVA followed by a Scheffe’s *f*-test by using SPSS 17.0 software (SPSS Inc., Chicago, IL, USA). Significance was chosen as *p* < 0.05.

## 4. Conclusions

FLZ prevents H_2_O_2_-induced RPE cell apoptosis through activating EGFR-AKT signaling. Since AMD is characterized by a progressive decay of RPE cells at the posterior pole of the eye and ROS are the major contributors of RPE damages in AMD, our results suggest that FLZ might have therapeutic value for AMD.
